# Independent Doctors

**Published:** 1878-04

**Authors:** 


### Independent Doctors.

A pleasing evidence that the "world moves," was the resolution of a [illegible]umber of prominent homeopathic physicians in England not to adhere [illegible]xclusively to the teachings of Hahnemann, but to employ any remedies [illegible]hat have been found useful as curative agents. That resolution has been [illegible]ndorsed by a convention of homoepathic physicians in this country. It [illegible] certainly creditable to their good sense. Medical science should be [illegible]rogressive. Although educated in the strictest school of allopathic med[illegible]cine, we have appropriated such remedies as our own experience has [illegible]roved to be the most prompt and effective in curing diseases. The strong [illegible]nan writhing in pain; the mother watching in tremulous anxiety over the [illegible]rostrate form of her suffering child, have no heart or interest in fine [illegible]heories; they demand relief, prompt and rational. It matters not if it comes from sugar pellets, from the bitter preparations of the druggist, or [illegible]rom patent medicines. The best remedy we have ever employed in [illegible]holera infantum and all bowel troubles of children is "homoepathic" in [illegible]aste and appearance at least. The only remedy that offers a reasonable [illegible]hance of a permanent cure for "constipation" is an advertised remedy. [illegible]or the encouragement of sufferers from constipation or from piles, we venture to print the following letter from a distinguished clergyman.

North Greenwich, Ct., March 25, 1878.

Dear Doctor—The "Nelaton's Suppositories" you so kindly had sent [illegible]o me, came promptly, and I immediately began using them according to [illegible]irection. Their effect was immediate. They acted like a charm. By [illegible]sing one every night a movement of the bowels was secured daily. I [illegible]ersevered in their use until I had used ten of them. I then thought best [illegible]o suspend their use, and though several days have elapsed, my bowels [illegible]ave moved regularly every day, which is a very novel and delightful [illegible]xperience after so many years of suffering I am under great obligations [illegible]o you for calling my attention to this remedy. Truly yours, A. Winter.

While on the subject of medicines, we cannot refrain from printing one [illegible]ore letter, which refers to another of our favorite remedies.

Waverly Station P. R. R., March 12, 1878.

[illegible]ditor of "Hall's Journal of Health," Bible House, New York.

Dear Sir—Noticing an advertisement of "Dr. Hall's Complete Cure for [illegible]ever and Ague" in the Journal, I have a word to say in its favor.

My wife and myself having suffered with fever and ague for some time without obtaining relief from any of the standard remedies, tried about [illegible]ne year ago, Dr. Hall's Cure with perfect success. I have recommended [illegible] to several friends, and have not heard of a single case of fever and ague [illegible]hich it failed to completely cure.

The following extract from a letter from George Chambers, Kinkora, [illegible]. J., to whom I sent a box of the "Cure," I heartily indorse.

"The pills you sent me, to the surprise of all my acquaintances as well as to myself, have completely cured me. I had suffered most of the time for [illegible]wo years, and for five months before taking Hall's cure I had hardly [illegible]issed a chill a day. Yours etc., J. C. Reid, Station Agent."

[illegible]We refer to this as one of our favorite remedies, because it contains [illegible]ther quinine or arsenic, and because we know from experience that it [illegible] an excellent remedy. We have prescribed it in hundreds of cases [illegible]uring the past year, and do not know that it has failed to cure immedi[illegible]tely and permanently in any instance. The price of each of these medicines is 50 cents a package. They will be sent by mail postpaid, on receipt of price by Hall & Ruckel, 218 Greenwich Street, New York.

